# The effects of spatial and temporal cueing on metacontrast masking

**DOI:** 10.2478/v10053-008-0093-1

**Published:** 2011-12-22

**Authors:** Maximilian Bruchmann, Philipp Hintze, Simon Mota

**Affiliations:** Institute for Biomagnetism and Biosignalanalysis, University of Muenster, Germany

**Keywords:** visual masking, metacontrast, spatial cueing, temporal cueing

## Abstract

We studied the effects of selective attention on metacontrast masking with 3 different cueing experiments. Experiments 1 and 2 compared central symbolic and peripheral spatial cues. For symbolic cues, we observed small attentional costs, that is, reduced visibility when the target appeared at an unexpected location, and attentional costs as well as benefits for peripheral cues. All these effects occurred exclusively at the late, ascending branch of the U-shaped metacontrast masking function, although the possibility exists that cueing effects at the early branch were obscured by a ceiling effect due to almost perfect visibility at short stimulus onset asynchronies (SOAs). In Experiment 3, we presented temporal cues that indicated when the target was likely to appear, not where. Here, we also observed cueing effects in the form of higher visibility when the target appeared at the expected point in time compared to when it appeared too early. However, these effects were not restricted to the late branch of the masking function, but enhanced visibility over the complete range of the masking function. Given these results we discuss a common effect for different types of spatial selective attention on metacontrast masking involving neural subsystems that are different from those involved in temporal attention.

## Introduction

Attending to a stimulus and becoming aware of it go hand in hand in everyday life. Yet, awareness and attention are not identical (Lamme, 2003). For example, it is known from patients suffering from lesions of their primary visual cortex that attention can have an effect on detec-ting stimuli in the patients’ blind visual field of which these patients remain unaware (*blindsight*; Kentridge, Heywood, & Weiskrantz, 1999; Kentridge, Nijboer, & Heywood, 2008).

In healthy subjects, awareness can be manipulated by employing metacontrast masking, which is one classical type of visual backward masking. Awareness of a briefly flashed target stimulus can be decreased or even completely prevented by the following presentation of a surrounding masking stimulus (for an overview, see e.g., Breitmeyer & Ögmen, 2000, 2006; Enns & Di Lollo, 2000). When visibility of the target stimulus is plotted against the stimulus onset asynchrony (SOA) of target and mask, one typically obtains a U-shaped masking function. It has been argued that the reason for a U-shape is the superposition of at least two processes (Michaels & Turvey, 1979; Reeves, 1982; Turvey, 1973): At the descending branch (SOA = 0 ms up to about 60 ms) subjects perceive target and mask as one stimulus whose visibility in the area of the target decreases while the visibility of the mask does not change substantially. Michaels and Turvey (1979, p. 1) called this *integration by common synthesis*, that is, due to their temporal proximity, target and mask “yield one iconic representation” comprising features of both stimuli. At the ascending branch of the masking function, subjects are progressively better able to detect a temporal separation of the two stimulus events. The visibility of the target increases monotonically with the likelihood with which targets and masks are perceived as separate events (Michaels & Turvey, 1979; Reeves, 1982).

 The dissociation of the descending and ascending part is mirrored in the effects of selective attention on metacontrast. Boyer and Ro (2007) used a version of Posner’s classical symbolic cueing paradigm (Posner, 1980) in which arrows are presented before the target-mask sequence. When arrows pointed to the correct position of the target, detection performance was increased (compared to when they pointed to the wrong position). This effect appeared exclusively at the ascending branch of the masking function. Similarly, Neumann and Scharlau (2007) found that presenting a distracting task-irrelevant stimulus contralateral to the target decreased target detectability at the ascending branch. Tata (2002) used peripheral cues in a metacontrast paradigm, that is, target position was indicated by a stimulus appearing at the exact location of the consecutive target and found increased detection rates if the target location was validly cued. Tata kept the SOA constant at 80 ms, which is typically in the range of the ascending branch of the masking function. In general, the finding that effects of attention do not simply counteract the awareness-reducing effects of metacontrast is further evidence that attention and awareness are qualitatively different concepts. 

The studies discussed above used either central symbolic or peri-pheral flanking cues to direct attention to the target location (or away from it). In none of the cited studies symbolic and flanking cues were compared in a single experiment, or in two otherwise comparable experiments. With the first two of the reported experiments we addressed this question. Symbolic and flanking cues were compared within the same paradigm. Note that we refrain from calling these cue types endogenous and exogenous cues, respectively, because exogenous cueing requires the cues to be uninformative (i.e., a ratio of 1:1 of valid and invalid cues; Carrasco, 2011) and in the present experiments we used informative cues, both in the symbolic and flanker cueing tasks. Compared to previous studies we further improved the design in two ways: We mo-nitored eye movements by means of an eye-tracker and we included a neutral cueing condition to be able to dissociate attentional benefits and costs. Differentiating between costs and benefits may allow conclusions about the way attentional resources are assigned to the visual input.

It further remains to be clarified whether enhanced visibility at the late branch of the masking function is only found when subjects attend to the correct *location* of the target. The third experiment extends the study of effects of attention on metacontrast by providing the subject with (valid or invalid) information not about where the target appears but *when* it will appear. Temporal cueing has been shown to enhance behavioral performance (Coull & Nobre, 1998; Nobre, 2001). Studies measuring event-related EEG potentials suggest that temporal cues facilitate performance by enhancing early visual processing steps (Correa, Lupiáńez, Madrid, & Tudela, 2006), especially if the task is perceptually demanding. With this comparison of the effects of three different types of selective attention on metacontrast masking we seek to clarify how specifically selective attention interacts with the modulation of conscious stimulus perception by visual masking.

## Experiments 1 and 2: Effects of central symbolic and peripheral flanking cues on metacontrast masking

 All reported experiments were adapted versions of the experimental design used by Bruchmann, Breitmeyer, and Pantev (2010). Targets and masks consisted of sinusoidal gratings with a Gaussian envelope. The participants’ task was to rate the visibility of the target subjectively on a 5-point scale. As reported recently by Albrecht, Klapötke, and Mattler (2010), subjects may show Type-A (i.e., monotonous) or Type-B (U-shaped) masking functions depending on their individual strategy. In our previous studies (Bruchmann et al., 2010), we had observed that with Gaussian blurred stimuli in combination with a subjective ratings task in which targets were presented on every trial (except for a few control trials), subjects did not appear to engage in different strategies. 

**Figure 1.**
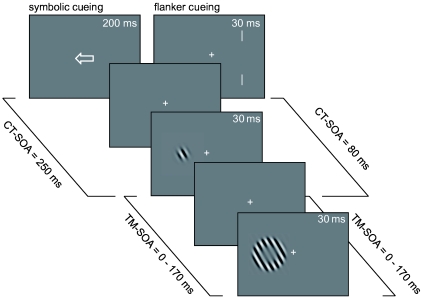
Trial sequences used in the symbolic and flanker cueing experiments. Symbolic cues were presented for 200 ms, flanker cues for 30 ms. The cues were followed by a blank interval of 50 ms, resulting in a cue-to-target-stimulus onset asynchrony (CT-SOA) of 250 ms and 80 ms, respectively. Cues could be valid, invalid, or neutral (double headed arrow / flankers on both sides) with a ratio of 3:1:1. Targets and masks were presented for 30 ms. On each trial the target-mask- stimulus onset asynchrony (TM-SOA) was chosen randomly between 0 and 170 ms.

Because of the similarity of both tasks we will first describe the design and methods of both and then report the results. Figure 1) shows exemplary trial sequences for the symbolic and flanker cueing experiments. For each of the two cue types we chose cue-to-target-SOAs (CT-SOAs) that are in the typical range reported in the literature (Carrasco, 2011; Cheal, Lyon, & Hubbard, 1991; Hein, Rolke, & Ulrich, 2006; Ling & Carrasco, 2006; Müller & Rabbitt, 1989). For symbolic cues we used a CT-SOA = 250 ms and for flanking cues we used a CT-SOA = 80 ms.

### Subjects

Six subjects (five female, one male) participated in both experiments. One half of the subjects started with the symbolic cueing experiment, and the other half with the flanker cueing experiment. All had normal or corrected to normal vision and no history of neurological or psychiatric diseases. Their age was 22 to 24 years (*M* = 23, *SD* = 0.7). Four subjects were right-handed, two left-handed. The subjects gave their informed consent and volunteered for participation and were paid 9 € per hour. All procedures were carried out according to the declaration of Helsinki and were approved by the ethical committee of the medical faculty of the University of Münster.

### Apparatus and stimuli

The experiment was run using SR Research Experiment Builder (SR Research Ltd., version 1.6.1). Stimuli were presented on a Samsung SyncMaster 1100P screen at a resolution of 1,024 × 768 pixel and 100 Hz, at a viewing distance of 65 cm. The subjects responded by pressing one of five buttons on an external response box. The participants were instructed to focus on a central fixation mark. To monitor the subject’s eye-movements, a head-based SR Research Ltd. EyeLink II eye-tracking device (version 2.22), was used. Once the focus deviated more than 2 degrees of visual angle (°) from the central fixation mark, a warning message was displayed and the respective trial was reintegrated into the condition list at a random point for later presentation. As target stimuli, Gabor patches with a diameter of 2° (measured from -2.5 to 2.5 *SD* of the Gaussian envelope) were used. As mask, a grating annulus with a Gaussian envelope was used. The diameter of the Gaussian envelope was 2°. Targets and masks were centered randomly 5° to the left or right of the fixation mark. Both had a spatial frequency of *f* =4 cycles per degree of visual angle (cpd) and were presented at six orientations: φ = 0°, 30°, 60°, 90°, 120°, and 150°. The phase of the sinusoidal luminance modulation was φ = 0° in the target and φ = 180° in the mask, meaning that each white “stripe” in the target was aligned with a black “stripe” of the mask and vice versa. The mask was presented at 100% black-and-white-contrast, the target at 90% black-and-white-contrast. The background color was middle gray. The symbolic cue stimulus was the white outline of an arrow, 3° in length. It could point either to the left or to the right or (in the neutral condition) could be double-headed, pointing in both directions. The flanker cue stimulus was a white line 2.5° above and below the possible location of the target stimulus. It could be presented at one or (in the neutral condition) at both locations.

### Symbolic cueing: Procedure

The subjects were instructed to focus on the fixation mark. Trials star-ted with the symbolic cue stimulus, presented for 200 ms. The interval between cue offset and target onset was 50 ms. The cue-to-target-SOA therefore was 250 ms. The cue could be valid (pointing to the correct side), invalid (pointing to the opposite side), or neutral (pointing to both sides) at a ratio of 3:1:1. The target was presented either to the left or to the right of the fixation mark for 30 ms, followed by the mask, also presented for 30 ms. The SOA between target and mask was either 0, 10, 20, 30, 40, 50, 60, 80, 120, or 170 ms.

Beside the target and mask conditions, we occasionally presented the target or the mask only. The target only conditions were supposed to “remind” subjects from time to time what they were supposed to detect. The mask only conditions were needed to obtain a false alarm rate (for details, see *Results* section). The orientation of the target and mask was varied randomly and averaged for the analysis. The resulting 72 experimental conditions (3 cueing conditions [valid, invalid, neutral] × 12 SOAs [10 SOAs + target-/mask-only trials]× 2 screen sides) were repeated 30 times in case of invalid and neutral trials and 90 times in case of valid trials, adding up to 3,600 trials. The trials were distributed over five sessions of 1 hr each. In order to get accustomed to the task, each subject performed 100 practice trials before response recording started. Subjects were asked to ignore the mask and to rate the visibility of the target stimulus after each trial, using one of five buttons, ranging from “0” = *not visible* to “4” = *clearly visible*. They were instructed to maintain a constant rating scheme over the experimental sessions and to use the full rating scale. The next trial started 200 ms after the response.

### Flanker cueing: Procedure

The procedure was equivalent to the previous experiment apart from the cue setup (see Figure 1)). The flanker cue stimulus could again be valid (presented on the correct side), invalid (presented on the opposite side), or neutral (presented on both sides simultaneously). The cue was presented for 30 ms and the onset asynchrony between cue and target stimulus was 80 ms. As in the symbolic cueing conditions the proportions of valid to invalid to neutral trials was 3:1:1.

### Results

 In order to exclude a possible response bias (e.g., deliberately giving higher ratings in valid trials), we chose not to analyze the raw rating data. Instead, we chose a signal detection theory approach (Green & Swets, 1966) where the visibility ratings are treated as detection data combined with a confidence rating (i.e., the lowest rating was treated as “target absent, high confidence”, the second lowest rating as “target absent, low confidence”, up to “target present, high confidence”). To obtain hit-rates (H), the relative frequency of each rating level in trials where a target was presented at a given SOA and cueing condition is first calculated and then summed over rating levels, yielding cumulative conditional probabilities. For *k* rating levels one obtains *k* – 1 cumulative hit rates, because the *k*^th^ level necessarily has a cumulative probability of *p* = 1. Similarly, the false alarm rates are calculated for each rating level in trials where only the mask was presented at a given cueing condition. Since there is no SOA in mask-only trials, the same false-alarm data is used for all SOAs in a given cueing condition. We then fitted a receiver operation characteristic (ROC) curve to the cumulative probabilities using the algorithm described by Dorfman and Berbaum (1986). For the ROCs we assumed a normal distribution of noise (i.e., internal activation in trials without a target) with μ_0_= 0 and σ_0_ = 1, and a normal distribution of signal + noise (i.e., internal activation in trials with targets) with μ_1_ and σ_1_ as free parameters. The analysis is based on the measure *Az*, that is, the area under the ROC curve (see e.g., Wickens, 2001) which ranges from *Az* = .5 for performance at chance level to *Az* = 1 for perfect detection. 

**Figure 2.**
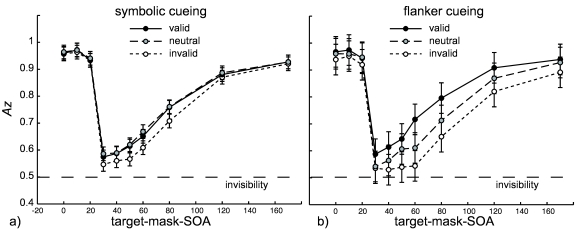
Averaged masking functions for the (a) symbolic and (b) flanker cue type. Error bars represent 95% confidence intervals for the effect Validity × SOA × Subject (see “Using Confidence Intervals in Within-Subject Designs” by G. R. Loftus and M. E. J. Masson, 1994, *Psychonomic Bulletin & Review, 1*, 476-490). SOA = stimulus onset asynchrony.

The averaged masking functions for valid, neutral, and invalid trials are shown in Figure 2a) for the symbolic cueing experiment and in Figure 2b for the flanker cueing experiment. We then performed a 3 (Validity) × 10 (SOA) ANOVA for repeated measurements, separately for cueing types. Reported p values are Greenhouse-Geisser corrected where necessary, or where sphericity assumptions could not be checked due to the low subjects-to-factor-levels ratio.

#### Symbolic cueing

For symbolic cues, we observed a significant main effect of SOA, *F*(9, 45) = 35.8, *p* < .001, no significant main effect of Validity, *F*(2, 10) = 2.0, *p* = .204, and no significant interaction between SOA and Validity, *F*(18, 90) = 1.1, *p* = .385.

To compare cueing effects on the early and late branch of the masking function, we calculated planned comparisons of valid and neutral conditions (benefits) and of invalid and neutral conditions (costs), separately for the averaged visibility at the early and late branch. To keep tests on the early and late branch equal in test power, we chose an equal amount of SOAs to test. The early branch was defined as SOAs 0 to 30 ms. The late branch was defined as SOAs 40 to 80 ms.

The planned comparisons for costs and benefits at the early part of the masking function yielded no significant costs (*p* = .155) or benefits (*p* = .630). On the late branch we found significant cost effects (*p* = .049) but no benefits (*p* = .700). Note that defining the late branch as ranging from 50 to 120 ms would have yielded higher statistical effects for costs, whereas defining it from 60 to 170 ms would not have yielded statistically significant effects, most likely due to the fully restored visibility at 170 ms. An a-priori definition of the exact SOAs defining the two branches was not possible for us, because the position of the masking function’s minimum is subject to many factors (e.g., stimulus contrast, eccentricity, etc.), and as such not precisely predictable.

#### Flanker cueing

For flanker cues we found a significant main effect of SOA, *F*(9, 45) = 74.5, *p* < .001, a significant main effect of Validity, *F*(2, 10) = 11.1, *p* < .001, as well as a significant interaction between SOA and Validity, *F*(18, 90) = 6.4, *p* < .001.

Planned comparisons as described above revealed that at the early branch we did observe neither significant benefits (*p* = .246), nor costs (*p* = .386). At the late branch we can report significant benefits (*p* = .0247) and marginally significant costs (*p* = .060).

### Discussion

 The results on symbolic cueing effects partly replicate those of Boyer and Ro (2007), as we also found significant differences after the SOA of optimal masking. In addition, we see that the cueing effect is completely due to attentional costs rather than benefits, that is, compared to the neutral condition, visibility is not enhanced when arrows indicate the correct location of the target, but visibility is reduced when arrows point to the wrong location. The effect is small, similar to Boyer and Ro’s result. Flanker cues exhibit a larger effect on the masking function. We observed attentional costs as well as benefits. In all cases, effects were restricted to the late branch of the masking function. To compare the effects of both experiments we calculated an additional 2 (Experiment) × 3 (Validity) × 10 (SOA) ANOVA. As expected, the three-way interaction was significant, *F*(18, 90) = 2.3, *p* = .005, confirming that flanker cueing effects were substantially larger than symbolic cueing effects. 

It may be argued that effects at the early part of the masking function could have been obscured by a ceiling effect at SOAs = 0 to 20 ms and that intermediate levels of visibility would have obtained between SOAs of 20 and 30 ms. Due to the monitor refresh rate we were bound to a spacing of SOAs by at least 10 ms. Thus, we were not able to cover the early branch in more detail. To check for a possible ceiling effect we analyzed the inter-individual variation in visibility at the early part (i.e., the individual *Az* averaged over Validity and SOAs of 0 to 20 ms) and correlated this measure with the attentional costs and benefits (also ave-raged over SOAs of 0 to 20 ms). Given a substantial variation in average visibility, a ceiling effect would imply a negative correlation of visibility and the negative or positive effects of cueing. The observed range of averaged visibilities was *Az* = 0.935 to *Az* = 0.980. For symbolic cueing, the Pearson-correlation coefficient for cueing effects and averaged visibility was positive but statistically nonsignificant for costs (*r* = .554, *p* = .254), and negative but also nonsignificant for benefits (*r* = .391, p = .444). For flanker cueing, the correlation was negative but statistically nonsignificant for costs (*r* = .281, *p* = .590) as well as for benefits (*r* = .355, *p* = .490). Since the inter-individual variation of averaged *Az* at the early part of the masking function as well as the sample size were small, we have to acknowledge that cueing effects at the early part of the masking function cannot be excluded based on our present data.

## Experiments 3: Effects of temporal cueing on metacontrast masking

**Figure 3.**
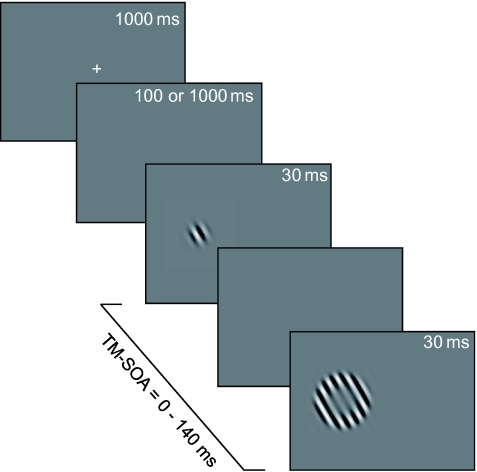
Trial sequences used in the temporal cueing experiment. Cueing the time point of target occurrence was achieved by introducing an interval between fixation mark offset and target onset with two fixed durations, t_1_ and t_2_, where t_1_ was eight times more frequent than t_2_. After a short learning period subjects began to expect target occurrence after t_1_. The interval lengths used for t_1_ and t_2_ were 100 ms and 1 s. Subjects completed two sessions of the experiment, with 100 ms as t_1_ in one session and as t_2_ in the other. TM-SOA = target-mask- stimulus onset asynchrony.

 In this experiment, the offset of the fixation mark was used as a temporal cue (see Figure 3). Over the course of the experiment, subjects were supposed to learn that in most cases the offset of the fixation mark preceded the target onset by a fixed temporal interval (t_1_). In the remaining trials the subjects’ expectation was violated and the target was presented after a different temporal interval (t_2_). This procedure was first described by Coull and Nobre (1998). It is well known that subjects intuitively establish an accurate representation of the frequency of events even if not instructed to do so (for a review, see Hasher & Zacks, 1984). The experiment consisted of two sessions between which the values for t_1_ and t_2_ were exchanged. 

### Subjects

Nine subjects (five female, four male) participated in the experiment. All had normal or corrected to normal vision and no history of neurological or psychiatric diseases. Their age was between 22 and 29 years (*M* = 25, *SD* = 2.52). Seven subjects were right-handed, two left-handed. The subjects gave their informed consent and volunteered for participation and were paid 9 € per hour. All procedures were carried out according to the declaration of Helsinki and were approved by the ethical committee of the medical faculty of the University of Münster.

### Apparatus and stimuli

 The experiment was run using MATLAB and the PsychophysicsToolbox (Brainard, 1997). Stimuli were presented on a ViewSonic G90fB CRT monitor at 100 Hz and a resolution of 1,024 × 768 pixels at a viewing distance of 80 cm. The mean brightness of the monitor was set to approximately 50 cd/m^2^ (I_min_ = 0.413 cd/m^2^, I_max_ = 100.201 cd/m^2^). Participants gave their responses by pressing one of four buttons on an external response box. The stimuli were generated as described by Bruchmann et al. (2010). All stimuli were always presented at the ma-ximum Michelson contrast of (I_max_ - I_min_) / (I_max_ + I_min_) = 0.992. Stimulus dimensions were identical to those in the two previous experiments. Targets and masks had a spatial frequency of *f* =2 cpd and were presented at random orientation, with the target and mask always sharing the same orientation. 

### Procedure

The general procedure (i.e., stimulus durations and dimensions, SOA randomization, control trials with target or mask only) was identical to the previous experiments. The fixation mark was shown for 1 s before it disappeared. The target-mask sequence appeared at random to the left or the right of the fixation mark after one of two possible CT-SOAs. The CT-SOAs were 100 ms and 1 s. In each of two experimental sessions per subject one was used eight times more often than the other (validity 8:1). The order of sessions was balanced across subjects. SOA varied randomly between 0, 30, 50, 60, 80, 110, and 140 ms. In each session, the invalid condition comprised 180 trials (2 sides × [7 SOAs + 1 target only reference trial + 1 mask only reference trial] × 10 re-petitions). The valid condition was eight times more frequent than the invalid, yielding 1,440 trials. In each of two experimental sessions of 90 min, participants completed 1,620 trials. After each trial, the participants were asked to rate the visibility of the target with four buttons. They were instructed to press button “1” if the target was not visible at all, and button “4” if it was well visible, and to use buttons “2” and “3” for intermediate visibility. The participants were asked to try using the full rating scale and to establish a constant rating scheme. Before starting the main experiment, the participants had 5 min of training to get familiar with the task.

### Results

Again, we calculated the sensitivity index *Az* from the relative frequencies of each rating level for masked targets and mask-only trials. We then performed a 2 (Interval Lengths) × 2 (Validity) × 7 (SOA) ANOVA for repeated measurements. The assumption of sphericity, as tested by the Mauchly Sphericity Test, was violated for the factor SOA, χ^2^(20) = 69.9, *p* < .001. Reported p values are Greenhouse-Geisser corrected where necessary.

We observed a significant main effect of SOA, *F*(6, 48) = 30.8, *p* < .001, a significant main effect of Interval Length, *F*(1, 8) = 15.0, *p* = .005, and no significant main effect of Validity, *p* = .086. The two-way interaction Interval Length × SOA was significant, *F*(6, 48) = 5.5, *p* = .047, as well as the three way interaction Interval Length × SOA × Validity, *F*(6, 48) = 2.5, *p* = .037. To resolve the three-way interaction we ran separate ANOVAs for each interval length with the factors SOA and Validity. For the short interval, we observed a significant main effect of SOA, *F*(6, 48) = 21.5, *p* < .001, and a significant main effect of Validity, *F*(1, 8) = 7.7, *p* = .024. The interaction SOA × Validity was not significant (*p* = .351).

**Figure 4.**
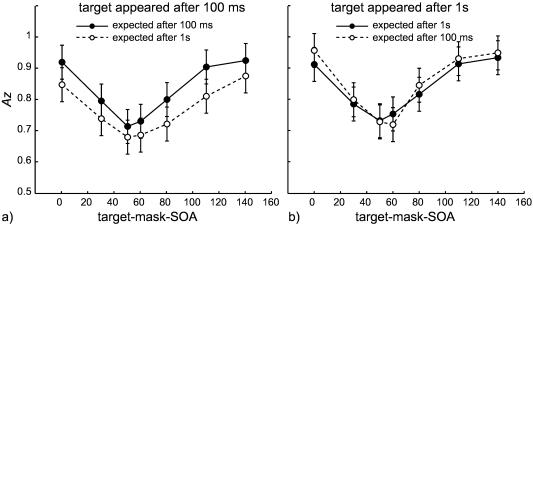
Averaged masking functions for the temporal cueing experiment. Figure 4a shows the two masking functions for the targets appearing after 100 ms, Figure 4b − for targets appearing after 1 s. In both figures the solid line with black circles shows the condition where the subjects expected the target at the point in time where it actually appeared. The dashed line with white circles depicts the performance in trials where the subjects expected the target at a different point in time. Note that each plot contains masking functions generated from two different sessions, that is, the solid line of one plot and the dashed line of the other belong to the two conditions recorded in one session. Error bars represent 95% confidence intervals for the effect Interval Length × SOA × Validity × Subject (see “Using Confidence Intervals in Within-Subject Designs” by G. R. Loftus and M. E. J. Masson, 1994, *Psychonomic Bulletin & Review, 1*, 476-490). SOA = stimulus onset asynchrony.

For the long interval, we found again a significant effect of SOA, *F*(6, 48) = 25.2, *p* < .001. In contrast to the short interval there was no effect of Validity (*p* = .521) but instead a significant SOA × Validity interaction, *F*(6, 48) = 2.9, *p* = .047. Post-hoc comparisons at each SOA for “valid vs. invalid” with Tukey-tests corrected for multiple application were all not significant (all *ps* ≥ .155, with the smallest p value observed at SOA = 0 ms), and could thus not provide certainty about the reason for the interaction. Descriptively, we observed slightly higher visibility in invalid trials at short SOAs (0 and 30 ms) and long SOAs (80, 110, and 140 ms). At intermediate SOAs, this difference was either not observable (SOA = 50 ms), or reversed (SOA = 60 ms, see Figure 4b).

To compare cueing effects on the early and late branch of the masking function, we calculated planned comparisons of valid and invalid conditions, separately for the averaged visibility of SOAs between 0 and 50 ms (early branch) and SOAs between 60 and 120 ms (late branch). Again, defining the ascending branch as 80 to 140 ms would have been less conservative. Since the interaction of SOA and Validity is only present for the long interval, the statistics actually do not justify a separate look at the two branches in the short interval. Nevertheless, we provide the results for the sake of completeness.

For targets presented after 100 ms, there was no significant cueing effect at the early branch (*p* = .085). At the late branch target visibility was significantly higher if the temporal cue was valid (*p* = .016). For targets presented after 1 s, there were no significant effects of cueing at the early (*p* = .388) or late (*p* = .692) branch.

### Discussion

 In contrast to the effects of spatial cueing (peripheral or symbolic), temporal cueing can affect the complete masking function. We observed higher visibility ratings for targets appearing after 100 ms when the target was expected to appear at this time point compared to when it was expected to appear after 1 s. This effect was not found for targets appearing after 1 s. A similar finding was reported by Coull and Nobre (1998) : In their temporal cueing study they observed validity effects in all conditions, except for those in which temporal cues incorrectly predicted the target’s appearance at the long time interval. As we did, the authors found no deleterious effect when the subject expected the target to occur at the short time interval but it actually occurred at the longer one. The lack of a cueing effect was explained by a “reorientation of attention” toward the long CT-SOA (Coull & Nobre, 1998). Since subjects learned that only two intervals were used, omission of the target at the short interval guaranteed it would occur at the long interval. In line with this interpretation is the observation that the masking functions for targets appearing after 1 s have the same shape as the function for targets presented and expected after 100 ms and not as the function presented but not expected after 100 ms. We conclude that under all conditions, except when targets appeared unexpectedly early, attention was present at the moment the target appeared. 

Our results further indicate that, depending on SOA, reorienting attention from the short to the long interval may even increase visibility as compared to a direct shift towards the long interval. This is indicated by the SOA × Validity interaction for stimuli presented after 1 s in combination with the descriptively higher visibility ratings at short and long SOAs in invalid trails compared to valid trials. However, the present data are insufficient to clarify whether benefits of a temporal reorientation of attention exist and in how far they are modulated by SOA.

## General Discussion

Three different types of attentional cues were used to study the effects of selective attention on metacontrast masking. Both spatial cue types revealed that expecting targets at the wrong location reduced target visibility exclusively at the late branch of the masking function. Additionally, flanker cues, but not symbolic cues, provided attentional benefits when the correct location was attended, again only at the late branch. Temporal cues provided a different picture: Expecting targets later than they actually appear yielded decreased visibility ratings, irrespective of SOA. Expecting targets earlier than they actually appear, did not lower or lift the masking function as a whole. There appeared to be subtle variations of a cueing effect with SOA, indicating that at short and long SOAs there was also a benefit from reorienting temporal attention after the expectancy of an early target had been violated.

The symbolic cues in this study match the classic definition of endogenous cues, which means that they are assumed to trigger a slow, voluntary shift of attention to the cued location. The flanker cues do not match the classic definition of exogenous cues since they were informative. Thus, we cannot exclude that the flankers triggered fast involuntary as well as slow voluntary attentional shifts. However, endogenous attention takes on average about 300 ms to develop its full effect (Carrasco, 2011). Since the CT-SOA used for flankers was 80 ms, we conclude that the major attentional resources contributing to the observed effects stem from a fast involuntary attentional system.

Interestingly, we observed qualitatively comparable effects of symbolic and flanker cues on metacontrast masking although they can be assumed to trigger fundamentally different mechanisms of attention allocation. The difference is merely that flanker cueing effects are larger and reflect attentional costs as well as benefits, whereas symbolic cueing effects are smaller and appear to reflect only attentional costs.

 The conditions under which selective attention is proposed to have an effect are discussed below. A model dealing with the question how selective attention may affect visual masking was proposed by Smith and colleagues (Smith, 2000; Smith, Lee, Wolfgang, & Ratcliff, 2009; Smith & Wolfgang, 2004). The authors propose that the mask limits the time target information is represented at a sensorial processing level. The allocation of attention to the target area causes an increase of the speed with which sensory information is read out to short-term memory. With the present results we can add to this model that symbolic and flanker cues appear to have comparable effects, except that only with flankers we found that a valid cue is better than a neutral cue. In Smith et al.’s model, *attention* is described as a spatiotemporal filter, which corresponds to the classic spotlight metaphor of attention, with the exception that it is defined by three dimensions: a spatial dimension, an intensity dimension, and a temporal dimension. To explain the differences between symbolic and flanker cueing effects we draw on the finding that size and shape of the attentional spotlight can be influenced by the type of the cue (Castiello & Umiltŕ, 1990; Eriksen & St James, 1986; Eriksen & Yeh, 1985; Galera & Grünau, 2003). We assume that the attentional spotlight triggered by symbolic cues is comparably broad in the spatial dimensions, so that with neutral cues both locations share some attentional gain. Invalid cues shift the broad focus to the wrong side, leaving the target side unattended. Valid cues, however, do not provide significantly more gain than the neutral cue, due to the broad spatial distribution of attentional resources. In contrast, flanker cues trigger a spatially sharply focused attentional spotlight. A neutral cue may provide the same mild attentional gain as a neutral symbolic cue, but the other cues appear to allocate sharply focused attentional resources at the cued side, withdrawing resources from the uncued side. Castiello and Umiltŕ (1990) presented evidence for a trade-off between size and efficiency of the attentional spotlight. Hence, we observe not only larger effects with flanker cues, but attentional costs as well as benefits. All spatial cues provide sharp temporal information since the CT-SOA was fixed. 

 An explanation for the observation that only the late branch of the masking function is affected by spatial selective attention cannot be easily deduced from current theories or models of metacontrast masking. In the classic sustained-transient model (ST-model) by Breitmeyer and Ganz (1976), metacontrast is a consequence of the interaction between the delayed sustained signal of the target and the quick transient signal of the mask. Due to the timing difference of sustained and transient channels it takes a positive non-zero SOA for a maximum overlap, and hence maximum inhibitory effects, of target and mask signals. However, if we look at two points in the masking function that are equal in visibility, but one left of the masking maximum and one right of it (i.e., one point on the early branch, the other on the late branch), the ST-model proposes that the reason for incomplete visibility reduction is the same in both cases: Partial temporal overlap of sustained target signals with transient mask signals. Yet, we see that only to the right of the masking maximum visibility is affected by spatial selective attention. Other models, such as the lateral inhibition theory (Macknik & Livingstone, 1998; Macknik & Martinez-Conde, 2007) also do not propose different mechanisms to explain partial masking at the two branches. A distinction between the two branches is made by Michaels and Turvey (1979), by Turvey (1973), and by Reeves (1982). Although the authors do not fully agree concerning the exact number and types of processes responsible for the U-shaped masking function, they do agree that the late branch is characterized by temporal separability of target and mask appearance. Specifically, it has been shown that for SOAs beyond the SOA of maximum masking, the likelihood of perceiving two events is higher than perceiving a single event (Michaels & Turvey, 1979). Temporal separability may thus be the precondition for any effect of spatial selective attention to take place. Because the experimental task is to rate target visibility and to ignore the mask, we assume that the attentional focus adheres to the target only, not to the mask, and not to an integrated target-mask-object. 

The ST-model’s successor, the RECOD-model (Breitmeyer & Ömen, 2006), incorporates non-linear feedback loops to explain me-tacontrast masking. In this model, the late branch is characterized by an increasing number of uninterrupted re-entrant activity from higher to lower visual processing stages. This top-down directed information is likely to be the carrier of attentional information.

 All these explanations may be also valid for the effect of temporal cueing on the late branch of the masking function. However, the effect of temporal cueing (with targets appearing after 100 ms) was not limi-ted to the late branch. In our view, this indicates a qualitative difference between spatial and temporal attention. In general, this assumption is in line with Nobre’s (2001) review comparing spatial and temporal attention, where the author concludes that the mechanisms behind the two types are “not simply the same and redundant” (p. 1319). Our interpretation of the present result is that spatial attention interacts with, or modulates the target-mask interactions that are causing the meta-contrast phenomenon. Temporal attention, on the other hand, appears to have an additive effect on target visibility and may involve neural mechanisms or subsystems that are independent of those engaged in metacontrast. This hypothesis is supported by neuroimaging results on metacontrast on the one hand and spatial or temporal attention on the other: An fMRI study by Haynes, Driver, and Rees (2005) suggests that visibility reductions by metacontrast coincide with reduced effective connectivity between primary visual cortex and the fusiform gyrus (FG). FG has been repeatedly shown to be involved in spatial attention (Heinze et al., 1994; Hopfinger, Buonocore, & Mangun, 2000). Neural correlates of temporal attention, on the other hand, as observed by fMRI (Coull, Frith, Büchel, & Nobre, 2000; Coull & Nobre, 1998) or PET (Coull & Nobre, 1998), do not involve FG. Of course, we have to assume that numerous brain areas are engaged in metacontrast and spatial attention, and that even more brain areas are not involved in temporal attention. Consequently, showing that neural correlates of the former two share one brain area that the third one does not share cannot be treated as proof for FG being the neural locus at which spatial attention modulates the effectiveness of metacontrast. However, FG qualifies as a candidate for such a locus. 

 We conclude that spatial and temporal attention exhibit qualitatively different effects on metacontrast masking. Spatial cues leave the early branch of the metacontrast masking function unchanged, whereas temporal cues do not. Given the subtle and not yet clarified interaction of temporal cueing and metacontrast with targets appearing after 1 s, future experiments have to clarify the role of the exact choice of temporal intervals. Nobre (2001) discusses how not only the absolute duration of cue-target intervals but also the difference between the chosen intervals influences the size of the observed attentional effect on choice reaction time. In combination with metacontrast these interactions may be even more complicated, as indicated by our present results. We believe it to be a promising approach to study these interactions in more detail in order to learn more about the temporal relationships of stimulus processing and temporal attention. 
